# RETRACTION: *Trichosanthes kirilowii* Ethanol Extract and Cucurbitacin D Inhibit Cell Growth and Induce Apoptosis through Inhibition of STAT3 Activity in Breast Cancer Cells

**DOI:** 10.1155/ecam/9894048

**Published:** 2026-02-22

**Authors:** Evidence-Based Complementary and Alternative Medicine

RETRACTION: S. R. Kim, H. S. Seo, H.‐S. Choi, et al. “*Trichosanthes kirilowii* Ethanol Extract and Cucurbitacin D Inhibit Cell Growth and Induce Apoptosis through Inhibition of STAT3 Activity in Breast Cancer Cells,” *Evidence-Based Complementary and Alternative Medicine* 2013, no. 1 (2013): 975350, https://doi.org/10.1155/2013/975350.

The above article, published online on 30 September 2013 in Wiley Online Library (https://wileyonlinelibrary.com), has been retracted by John Wiley & Sons Ltd. The retraction has been agreed after an investigation into concerns raised by *Archasia belfragei* and *Illex illecebrosus* via PubPeer [1]. Specifically, the LC‐MS chromatogram in Figure 6a shares unexpected similarities with the mass spectrum in Figure 6b.

Further investigation also revealed unexpected similarities between the western blot band depicting Actin in Figure 3b and the band depicting Tubulin in Figure 4a.

Several attempts by the journal to contact the authors have not resulted in a response. As such, the results cannot be considered reliable.
